# Subtilase SprP exerts pleiotropic effects in *Pseudomonas aeruginosa*

**DOI:** 10.1002/mbo3.150

**Published:** 2013-12-26

**Authors:** Alexander Pelzer, Tino Polen, Horst Funken, Frank Rosenau, Susanne Wilhelm, Michael Bott, Karl-Erich Jaeger

**Affiliations:** 1 Institute of Molecular Enzyme Technology, Research Centre Juelich, Heinrich-Heine-University DuesseldorfD-52426, Juelich, Germany; 2 Institut of Bio-und Geosciences IBG-1: Biotechnology, Research Centre JuelichD-52426, Juelich, Germany; 3 Institute of Pharmaceutical Biotechnology, Ulm-UniversityAlbert-Einstein-Allee 11, D-89069, Ulm, Germany

**Keywords:** Biofilm, microarray, motility, orf PA1242, protease, *Pseudomonas aeruginosa*, Pyoverdine

## Abstract

The open reading frame *PA1242* in the genome of *Pseudomonas aeruginosa* PAO1 encodes a putative protease belonging to the peptidase S8 family of subtilases. The respective enzyme termed SprP consists of an N-terminal signal peptide and a so-called S8 domain linked by a domain of unknown function (DUF). Presumably, this DUF domain defines a discrete class of *Pseudomonas* proteins as homologous domains can be identified almost exclusively in proteins of the genus *Pseudomonas*. The *sprP* gene was expressed in *Escherichia coli* and proteolytic activity was demonstrated. A *P. aeruginosa* Δ*sprP* mutant was constructed and its gene expression pattern compared to the wild-type strain by genome microarray analysis revealing altered expression levels of 218 genes. Apparently, SprP is involved in regulation of a variety of different cellular processes in *P. aeruginosa* including pyoverdine synthesis, denitrification, the formation of cell aggregates, and of biofilms.

## Introduction

*Pseudomonas aeruginosa* is an opportunistic aerobic Gram-negative bacterium regarded as the major cause of death in cystic fibrosis patients (Murray et al. 2007). It causes both community-and hospital-acquired infections including ulcerative keratitis, skin and soft tissue infections, pneumonia, and infection after burn injuries (Zegans et al. 2002; Fujitani et al. 2011; Rezaei et al. 2011; Wu et al. 2011) and is the leading cause of respiratory tract infections in patients intubated during surgery, with a high mortality rate (Chastre and Fagon 2002). Epidemic increases in bacterial multidrug resistance and the occurrence of truly pan-resistant Gram-negative pathogens (e.g., *P. aeruginosa*, *Acinetobacter baumannii*, *Klebsiella pneumonia*) are major concerns in clinical microbiology, and thus, novel antibiotics are urgently needed (Payne 2008; Rasko and Sperandio 2010). However, the identification of potential drug targets and the success of novel antimicrobial agents proves to be difficult (Maeda et al. 2012). Part of the *P. aeruginosa* pathogenic potential is an impressive arsenal of virulence factors, like flagella and type IV pili, exopolysaccharides, lipopolysaccharides, and several secreted factors (Ramphal and Pier 1985; Nicas and Iglewski 1986; Drake and Montie 1988; Sato et al. 1988; Gupta et al. 1994). All these virulence factors are regulated by several complex regulatory systems including the quorum sensing (QS) system (Pesci et al. 1999; Venturi 2006). The yellow–green siderophore pyoverdine is also required for pathogenicity of *P. aeruginosa* as it is part of the major iron uptake system which is controlled by the iron starvation (IS) sigma factor PvdS (Ochsner et al. 2002; Tiburzi et al. 2008). Several other virulence factors including the proteases PrpL and AprA, are also regulated by PvdS (Shigematsu et al. 2001; Wilderman et al. 2001; Lamont et al. 2002). Two different surface organelles, namely a single polar flagellum and polar type IV pili, are responsible for *P. aeruginosa* motility. In liquid environments, *P. aeruginosa* can swim using flagella, whereas the type IV pili enable twitching motility on solid surfaces (Henrichsen 1972; Mattick 2002). On semisolid surfaces, *P. aeruginosa* moves by swarming *via* flagella and type IV pili (Köhler et al. 2000). These cell surface structures are also involved in biofilm formation (O'Toole and Kolter 1998; Klausen et al. 2003). *P. aeruginosa* biofilms pose a significant problem in both industrial and medical settings. Biofilms can form on medical devices like intravenous catheters and contact lenses and they are causative for the high resistance of *P. aeruginosa* against many antibiotics as well as the host immune system (Zegans et al. 2002). Biofilms exist in a complex extracellular matrix consisting of DNA, polysaccharides, and proteins. Their formation is a highly regulated process depending, among other factors, on cell motility and complex QS systems stabilizing such biofilms (Harmsen et al. 2010; Ghafoor et al. 2011; Mikkelsen et al. 2011).

Proteases represent a very diverse group of hydrolases which are found in all kingdoms of life (Rawlings and Barrett 1994). In bacteria, they are often involved in essential regulatory processes, for example, via degradation of abnormally folded proteins or by controlling the intracellular amounts of sigma factors and chaperones (Gottesman 1996). They catalyze the cleavage of peptide bonds in proteins and either show high specificity for defined amino acid (aa) sequences, or unspecifically hydrolyze proteins to yield oligopeptides or aa. Serine proteases contain serine as the active site residue and are grouped into 13 clans in the MEROPS database (Rawlings et al. 2012). Among them, family S8 of peptidases, also known as “subtilisin-like” or “subtilase” family, harbors the serine endopeptidase subtilisin and related enzymes with a characteristic Asp/His/Ser catalytic triad and represents the second largest family of serine proteases (Siezen and Leunissen 1997; Siezen et al. 2007; Rawlings et al. 2012). Subtilases are found in archaea, bacteria, viruses, fungi, yeast, and higher eukaryotes (Siezen and Leunissen 1997; Bergeron et al. 2000; Antao and Malcata 2005; Poole et al. 2007). The majority of subtilases is secreted but several members are localized intracellularly (Siezen and Leunissen 1997). In general, subtilases play a key role in protein maturation processes and precursor processing (Siezen et al. 2007). Subtilases typically show a multi-domain structure containing a signal sequence for translocation, a pro-domain for maturation, and a protease domain (Siezen and Leunissen 1997). The N-terminal propeptide of subtilases usually acts as an intramolecular chaperone (Shinde and Inouye 1993) that ensures correct folding of the enzyme, inhibits premature proteolytic activity (Kojima et al. 1997), and is autocatalytically cleaved off during enzyme maturation (Gallagher et al. 1995; Yabuta et al. 2001).

We have identified in the genome sequence of *P. aeruginosa* PAO1 (Stover et al. 2000), the open reading frame *sprP* (*PA1242*) encoding a so far hypothetical protein with homology to enzymes of the subtilisin-like peptidase family S8. The predicted protein SprP contains a signal sequence and a peptidase S8 domain. We have demonstrated that SprP exhibits protease activity and further report on the role of SprP for various important cellular functions in *P. aeruginosa* including growth, cellular motility, biofilm formation, and pyoverdine production.

## Experimental Procedures

### Bacterial strains, plasmids, media, and culture conditions

The strains and plasmids used in this study are listed in Table 1. *Escherichia coli* DH5*α* was used as host for cloning and expression; *E. coli* S17-1 was used for conjugal transfer. All strains were grown in lysogeny broth (LB) medium (10 g/L tryptone, 5 g/L yeast extract, 10 g/L NaCl). For anaerobic growth conditions, 50 mmol/L KNO_3_ was added to the medium. Cultures grown for 16 h at 37°C in LB medium (volume: 10 mL) in Erlenmeyer flasks were used to inoculate main cultures (volume: 25 mL) to an initial optical density of OD_580nm_ = 0.1. Cultures were grown at 37°C in a rotating shaker at 150 rpm until they reached an optical density of OD_580nm_ = 2.5. Sodium nitroprusside (SNP) was added to LB-medium at a concentration of 2 mmol/L. For *E. coli*, chloramphenicol (50 *μ*g/mL) was used and for *P. aeruginosa*, chloramphenicol (300 *μ*g/mL), and gentamycin (30 *μ*g/mL) was added.

**Table 1:** Strains, plasmids, and primers used in this study.

Strain or plasmid	Genotype/Phenotype	Reference or source
Strains		
* Pseudomonas aeruginosa*		
PAO1	Wild type	Holloway et al. (1979)
Δ*sprP*	Δ*sprP* (*sprP*::Ω*Gm*^*r*^)	This work
* Escherichia coli*		
DH5*α*	*supE44 Δ*(*lacZYA-argF*)*U196* (*Δ80ΔlacZM15*) *hsdR17 recA1 endA1 gyrA96 thi-1 relA1*	Woodcock et al. (1989)
S17-1	Ec294::[RP4-2 (Tc::Mu) (Km::Tn7)], *pro, res, recA, tra*^*+*^, Tp^r^, Sm^r^	Simon et al. (1983)
Plasmids		
pBBR1MCS	Cm^r^ mob *lacZα* P_*lac*_ P_T7_	Kovach et al. (1994)
pSUP202	pBR325, Ap^r^ Cm^r^ Tc^r^ *mob*	Simon et al. (1983)
pBSL142	ColE1 Ap^r^ Gm^r^	Alexeyev et al. (1995)
pBCSK	P_37_ P_T3_ P_lac_ *lacZ*' Cm^r^ ColE1	Stratagene
pTZ110	Cb^r^; promoterless *lacZ*	Schweizer and Chuanchuen (2001)
Primer		Cloning site
* sprP_up*	CCGAAGCTTCCGGAAGCCGAGTCCATGCCG	*Hind*III
* sprP_dw*	AAGTGCGGATCCTCAGCGCACGCGCTC	*Bam*HI
* sprP*_lacZup	GGGAATTCGCGCCTGCGGCTTGA	*Eco*RI
* sprP*_lacZdown	AAGGATCCGGACTCGGCTTCCGGAAC	*Bam*HI
USTUP	AAATCTAGATGGAGCGGAAACTTCTAGTTA	*Xba*I
USTDW	GGGAATTCACGCGTGGACTCGGCTTCC	*Eco*RI
DSTUP	GGGAATTCACGCGTGGCGTTCCGCCGGCC	*Eco*RI
DSTDW	GGAAGCTTGGTGGTGGATCCGCACCAGTTCGA	*Hind*III

### Recombinant DNA techniques, gene cloning, and mutant construction

Recombinant DNA techniques were performed essentially as described by Sambrook et al. (1989). DNA fragments were amplified by polymerase chain reaction (PCR) standard methods. DNA modifying enzymes (Fermentas, St-Leon-Roth, Germany) were used according to manufacturer's instructions. Plasmid DNA was prepared as described by Birnboim and Doly (1979) and by using the innuPREP Plasmid Mini Kit (Analytik Jena, Jena, Germany) or, for genomic DNA from *P. aeruginosa*, the DNeasy Blood & Tissue Kit (Qiagen, Hilden, Germany).

The gene *sprP w*as amplified by PCR using primers *sprP*_up and *sprP*_dw (Table 1) and chromosomal DNA of *P. aeruginosa* PAO1 as the template. The PCR products were digested with *Hind*III and *Bam*HI, ligated into plasmid pBBR1MCS resulting in plasmid pBBRSP.

Construction of *sprP* deletion mutant was performed as described by Tielker et al. (2005). The plasmids for genomic deletion of *sprP* were constructed by PCR amplification of the regions located upstream and downstream of the *sprP* gene by use of the primers USTUP + USTDW and DSTUP + DSTDW. The resulting fragments were cloned into the *Xba*I/*Eco*RI and *Eco*RI/*Hind*III sites of plasmid pBCSK giving pUST and pDST, respectively. An Ω-gentamicin cassette was isolated as a 1.6 kb *Mlu*I fragment from plasmid pBSL142 and was subsequently cloned into the *Mlu*I site of pUST resulting in pUSTGm. The downstream fragment was then cloned into pUSTGm via *Eco*RI/*Hind*III to give pSPGm. pSPGm was digested with *Xba*I/*Hind*III and blunted with T4-polymerase. The fragment harboring the Ω-gentamicin cassette was ligated into *Sca*I-digested pSUP202 resulting in the suicide vector pSUSPGm. For the generation of the transcriptional fusion plasmid harboring the promoter region of *sprP* fused to a promoterless *lac*Z gene (encoding *β*-galactosidase), the upstream region of the *sprP* gene was amplified by use of the primers *sprP*_lacZup and *sprP*_lacZdw (Table 1). The fragment was digested with *Eco*RI/*Bam*HI and cloned into plasmid pTZ110. The resulting plasmid pTZ*sprP* contains the promoter region (556 bp) of *sprP* fused to the promoterless *lacZ* gene.

### Construction of a *P. aeruginosa* Δ*sprP*

A *P. aeruginosa sprP* negative mutant was constructed by successively using the plasmid pSUSPGm. Replacement of the chromosomal copy of *sprP* was achieved after conjugation and homologous recombination with pSUSPGm. The *sprP* gene was deleted after homologous recombination to create an in-frame deletion and resulted in *P. aeruginosa* Δ*sprP* as confirmed by PCR. For complementation studies, plasmids pBBR1MCS and pBBRSP were introduced into *P. aeruginosa* Δ*sprP* by biparental mating.

### Determination of protease activity

#### Agar plate assay

Bacteria grown as described above were plated on LB agar containing 3% (w/v) skim milk, plates were incubated for 16 h at 37°C and afterward overnight at 4°C to reduce bacterial growth. Clear zones (halos) around the colonies indicate protease activity.

#### Liquid assay

Whole cell extracts were prepared from cells grown to an OD_580nm_ = 3. Cells were centrifuged at 21,500*g* for 20 min, resuspended in 0.2 mol/L Tris-HCl buffer, pH 8 (final OD_580nm_ = 10) and sonicated for 2 × 4 min at 25% power. The substrate Suc-Ala-Ala-Pro-Phe-*para*-nitroanilide (Sigma-Aldrich, Seelze, Germany) was dissolved in 0.2 mol/L Tris-HCl buffer, pH 8, to a final concentration of 10 mmol/L (DelMar et al. 1979). A volume of 30 *μ*L of whole cell extract was added to 35 *μ*L of substrate solution, the reaction tubes were incubated for 16 h at 37°C, and the absorption was determined at 410 nm indicating the release of *para*-nitroanilide from the substrate.

### Determination of *β*-galactosidase activity

*Pseudomonas aeruginosa* PAO1 was transformed with plasmid pTZ*sprP* and promoter activity of *sprP* was monitored by determination of *β*-galactosidase activity according to the method of Miller (1972). The cultures were grown in 100 mL LB medium and cell growth (OD_580nm_) was determined. *β*-galactosidase activity was measured for each sample after cell lysis by sodium dodecyl sulfate (SDS)/chloroform permeabilization. After quenching of the reaction using 1 mol/L Na_2_CO_3_ and centrifugation to remove the cell debris, 1 mL of each sample was transferred to a cuvette and the absorption at 420 nm was determined.

### Motility assays

The agar plate assays to analyze swarming, swimming, and twitching motilities were performed as described before (O'Toole and Kolter 1998; Rashid and Kornberg 2000; Tremblay and Deziel 2008). M9 minimal broth medium was used for swimming analysis. The swim plates contained, per liter, 4 g glucose, 0.25 g MgSO_4_, 0.02 g CaCl_2_, 7 g Na_2_HPO_4_, 3 g KH_2_PO_4_, 0.5 g NaCl, 1 g NH_4_Cl, and 0.3% w/v select agar (Invitrogen, Darmstadt, Germany). Swim plates were inoculated with 5 *μ*L bacteria from overnight cultures (OD_580nm_ = 3) grown in LB medium at 37°C. Plates were then incubated at 37°C for 16 h. The swarm plates contained, per liter, 1.07 g NH_4_Cl, 2.14 g Na_2_HPO_4_ × H_2_O, 2.99 g KH_2_PO_4_, 0.5 g NaCl, 0.25 g MgSO_4_, 0.15 g CaCl_2_ × 2 H_2_O, 1.98 g glucose, 5 g casamino acids, and 5 g select agar. Plates were dried for 30 min before use, inoculated with 5 *μ*L bacteria from overnight cultures (OD_580nm_ = 3) and incubated at 37°C for 16 h. The LB medium used to analyze the twitching motility contained, per liter, 10 g tryptone, 5 g yeast extract, 10 g NaCl, and 1.5% w/v agar. Plates were inoculated from overnight cultures of bacteria that grew on LB plates (1.5% w/v agar) using a sharp toothpick stabbed through the LB agar onto the bottom of the petri dish. The zone of motility, visible at the interface between agar and petri dish, was documented after incubation for 48 h at 37°C.

### Aggregate formation assay

Different bacterial strains were transferred into 25 mL LB medium to an initial optical density of 0.1 (OD_580nm_) and the cultures were cultivated at 37°C until a cell density of OD_580nm_ = 2.5 was reached. The formed aggregates were documented using photography.

### Biofilm formation

The tests were adapted from the method originally described by O'Toole and Kolter (1998). Bacteria were grown for 16 h at 37°C in LB medium in 24-well microtiter-plates. Cells attached to the plates were subsequently stained by incubation with 200 *μ*L 1% crystal violet (CV) for 20 min. Unattached cells were removed by rinsing the wells with water. The water remaining in the wells was evaporated at room temperature. The biofilm formation was photo documented. After addition of 1 mL ethanol the absorption at 590 nm was determined to quantify biofilm formation.

### Quantification of pyoverdine production

*Pseudomonas aeruginosa* strains were grown in 10 mL LB medium to an OD_580nm_ = 2.5 and pyoverdine concentrations in cell-free culture supernatants, filtered with 0.22 *μ*m syringe filter (VWR International, Langenfeld, Germany), were determined as described (Meyer and Abdallah 1978).

### Anaerobic growth

*Pseudomonas aeruginosa* strains were grown anaerobically in 20 mL LB medium containing 50 mmol/L KNO_3_ as electron acceptor. Prior to inoculation, the Erlenmeyer flasks were rubber plugged and flushed with N_2_ to create an oxygen free atmosphere; residual oxygen is rapidly consumed by growing bacteria. As a control, cells were grown under identical conditions, but aerobically, that is without rubber-plugged flasks and exposure to N_2_. Cell growth was determined as OD_580nm_ during incubation at 37°C and rotary shaking after 6 h.

### DNA microarray analysis

#### Array preparation

DNA microarrays were obtained from Agilent Technologies (Waldbronn, Germany). Agilent's eArray platform was used to design oligonucleotide probes and assemble the custom 4 × 44 K 60mer microarray (https://earray.chem.agilent.com/earray/). For genome-wide gene expression analysis of *P. aeruginosa* PAO1, the cDNA sequences of the genome annotation from National Center for Biotechnology Information (NCBI) (NC_002516) listing 5571 protein coding genes and 106 structural RNA coding genes were used as input in eArray to design one 60-mer probe for each gene with the best probe methodology. Furthermore, the cDNA sequences representing the 200 bp upstream region relative to the annotated start of each gene was used to design one 60-mer probe for each gene using the best probe methodology. For *P. aeruginosa* PAO1, 5636 specific probes for the annotated genes and 5649 specific probes for the 200 bp upstream region of the annotated genes were designed by eArray. The custom array design also included Agilent's control spots.

#### cDNA synthesis and hybridization

*Pseudomonas aeruginosa* PAO1 and Δ*sprP* were grown in LB medium until cell growth reached an OD_580nm_ of 2.5. Total RNA was isolated by using the RNeasy kit (Qiagen) and treated with Ambion® DNase I (RNase-free) (Invitrogen, Darmstadt, Germany). cDNA synthesis for DNA microarray analysis was performed as described (Polen et al. 2007). A quantity of 25 *μ*g of RNA was used for random hexamer-primed synthesis of fluorescently labeled cDNA with the fluorescent nucleotide analogues Cy3-dUTP and Cy5-dUTP (GE Healthcare, Freiburg, Germany). The mixture contained 3 *μ*L of Cy3-dUTP or Cy5-dUTP (1 mmol/L), 3 *μ*L of DTT (100 mmol/L), 6 *μ*L of 5× first strand buffer (Invitrogen), 0.6 *μ*L of dNTP-mix (25 mmol/L each of dATP, dCTP, and dGTP and 10 mmol/L dTTP), and 2 *μ*L of Superscript II reverse transcriptase (Invitrogen, Darmstadt, Germany). The mixture was incubated for 10 min at room temperature and 110 min at 42°C, stopped by addition of 10 *μ*L of 0.1 N NaOH, incubated for 10 min at 70°C, and then neutralized by addition of 10 *μ*L of 0.1 N HCl. cDNA samples were purified by washing and centrifugation three times with water using Microcon columns (Merck, Darmstadt, Germany, YM-30). Purified cDNA samples to be compared were pooled and the prepared two-color samples (wild-type control vs. *sprP*-mutant strain) were hybridized at 65°C for 17 h using Agilent's Gene Expression Hybridization Kit and hybridization chamber. After hybridization the arrays were washed using Agilent's Wash Buffer Kit according to the manufacturer's instructions. Fluorescence of hybridized DNA microarrays was determined at 532 nm (Cy3-dUTP) and 635 nm (Cy5-dUTP) at 5 *μ*m resolution with a GenePix 4000B laser scanner and GenePix Pro 6.0 software (Molecular Devices, Sunnyvale, CA). Fluorescence images were saved to raw data files in TIFF format (GenePix Pro 7.0).

#### Data analysis

Quantitative TIFF image analysis was carried out using GenePix image analysis software and results were saved as GPR-file (GenePix Pro 7.0). For background correction of spot intensities, ratio calculation and ratio normalization, GPR-files were processed using the BioConductor R-packages limma and marray (http://www.bioconductor.org). For further analysis, the processed and loess-normalized data as well as detailed experimental information according to MIAME (Brazma 2009) were stored in the DNA microarray database of the Center of Molecular Biotechnology in the Forschungszentrum Jülich GmbH (Polen and Wendisch 2004). The DNA microarray analysis was repeated independently five times by biological replicates. To search the data for differentially expressed genes by the processed Cy5/Cy3 ratio reflecting the relative RNA level, the criteria flags ≥0 (GenePix Pro 7.0) and signal/noise ≥3 for Cy5 (F635Median/B635Median) or Cy3 (F532Median/B532Median) were used. If the signal/noise of Cy5 and of Cy3 were <3 then signals were considered as to weak to analyze the Cy5/Cy3 ratio of a gene. Furthermore, *P*-values were calculated by a paired Student's *t-*test comparing the relative RNA levels of a gene in the replicates to the relative RNA levels of all other genes in the replicates. In addition, we also calculated adjusted *P*-values according to the method of Benjamini and Hochberg (1995), which is implemented in the R-package limma in the p.adjust function.

### Computer analysis

The *sprP* gene sequence, the SprP protein sequence, and the downstream/upstream regions of *sprP* were retrieved from the pseudomonas.com database (Winsor et al. 2011). The prediction of the peptidase S8 domain and catalytic aa was performed with the conserved domain sequence (CDS) alignment search tool (Marchler-Bauer et al. 2011) offered by NCBI. The SprP protein sequence was used as a template and an E-value threshold of 0.01 was set. The signal peptide prediction was performed by using SignaP 4.0 (Petersen et al. 2011). General information about subtilisin-like serine proteases and protein classification was done with MEROPS (Rawlings et al. 2012) and the Prokaryote Subtilase Database (Siezen et al. 2007).

## Results

### SprP sequence analysis

**Figure 1:**
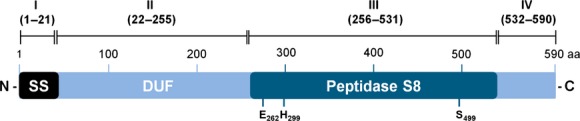
SprP domain composition. SprP consists of 590 amino acids (aa) with an N-terminal signal sequence (I, SS), a domain of unknown function (II, DUF), a peptidase S8 domain (III), and a C-terminal extension (IV). Numbers indicate the amino acids flanking the respective domains; putative catalytic triad residues E_262_, H_299_, and S_499_ are labeled.

A similarity search within the *P. aeruginosa* PAO1 genome using the aa sequence of *Bacillus licheniformis* subtilisin Carlsberg (Jacobs et al. 1985) revealed only two proteins with low but significant identities of 29% (PA1242) and 27% (EprS), respectively. These proteases were further analyzed for conserved domains (Marchler-Bauer et al. 2011) and orf PA1242 was identified to encode a putative protease previously annotated as a hypothetical protein in the *Pseudomonas* Genome Database (Winsor et al. 2011). It consists of 590 aa with a deduced molecular mass of ca. 64.9 kDa. The newly identified protein harbors an N-terminal type I export signal peptide of 21 aa followed by a 233 aa domain without any similarity to known proteins which was thus qualified as domain of unknown function (DUF). The C-terminal catalytic domain (aa 256–531) contains a putative protease motif of the MEROPS peptidase family S8 (Fig. 1). Given this similarity to the subtilisin-like family of proteases, we have named the protein encoded by PA1242 SprP (subtilisin-like protease P). A database search using the aa sequence of the DUF domain as template revealed 69 proteins with homology to hypothetical proteins and subtilases from various *Pseudomonas* species*,* namely *P. aeruginosa*, *Pseudomonas alcaliphila*, *Pseudomonas mendocina*, *Pseudomonas brassicacearum*, *Pseudomonas fluorescens*, *Pseudomonas poae*, *Pseudomonas putida*, *Pseudomonas fulva*, *Pseudomonas viridiflava*, and *Pseudomonas stutzeri* with identities varying from 100% to 53.4%. Interestingly, only three of the identified proteins belong to species other than *Pseudomonas:* a hypothetical protein from *Halomonas boliviensis* LC1 (58.7%), a predicted subtilase from *Halomonas sp*. HAL1 (58.6%) and a predicted subtilase from *Halomonas zhanjiangensis* (56.4%).

### SprP is a serine protease

**Figure 2:**
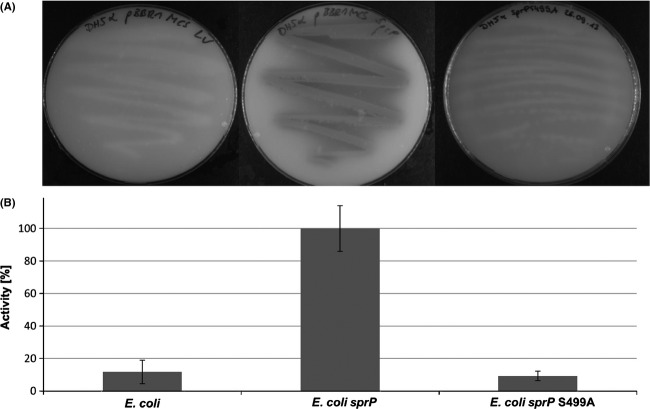
SprP is a protease. (A) *Escherichia coli* DH5*α* was transformed with plasmid pBBRSP harboring *sprP* (right) or empty vector (left), plated on agar containing 3% skim milk and plates were incubated for 16 h at 37°C. (B) Cell extracts of strains shown in (A) were prepared after centrifugation and subsequent sonication and tested for proteolytic activity with Suc-Ala-Ala-Pro-Phe-*p*-nitroanilide as the substrate. Relative activity of 100% corresponds to 0.308; error bars indicate standard deviation.

*Escherichia coli* cells harboring the *sprP* gene on plasmid pBBRSP were grown on agar plates containing skim milk as the protease substrate. Protease activity indicated by the formation of clear halos around the colonies was exclusively detectable with *E. coli* harboring the *sprP* expression plasmid whereas the vector control did not show halo formation. SprP variant S499A which had the catalytic serine replaced by alanine also did not show protease activity (Fig. 2A). Protease activity was quantified using the synthetic substrate *N*-succinyl–L-Ala–L-Ala–L-Pro–L-Phe–*p*-nitroanilide which is specifically hydrolyzed by serine proteases (Brode et al. 1996) (Fig. 2B).

### The sprP gene is expressed during onset of the stationary phase

**Figure 3:**
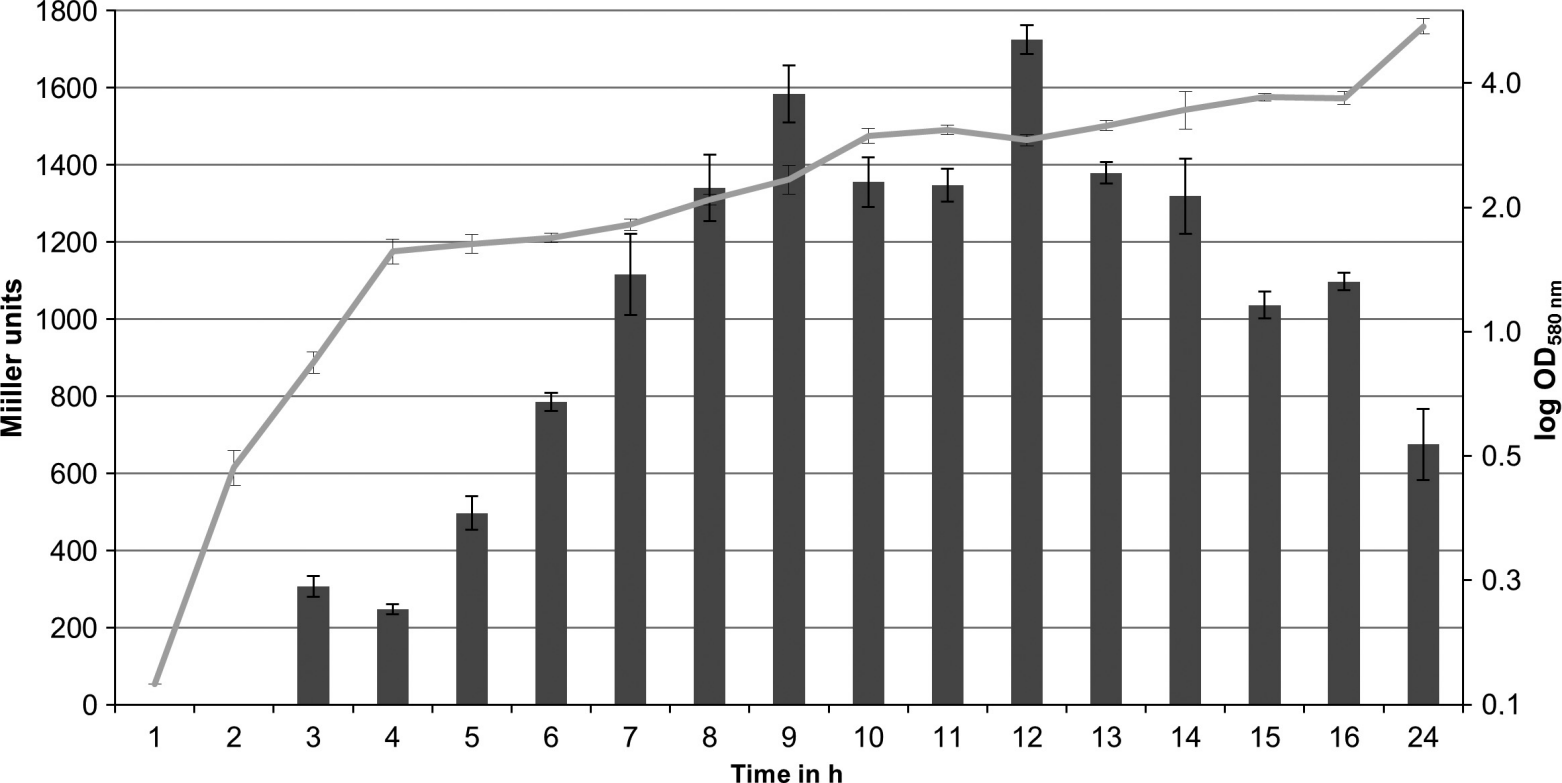
Promoter activity of *sprP*. *Pseudomonas aeruginosa* PAO1 containing plasmid pTZ*sprP,* which harbors a 556 bp DNA fragment upstream of *sprP* fused to *lacZ* was inoculated to an initial cell density of OD_580nm_ = 0.1 and incubated for 24 h at 37°C. Activity of *β*-galactosidase (bars) was determined as described by Miller (1972) and the corresponding growth of the culture (line) was determined as absorbance of the bacterial culture at 580 nm. Error bars indicate standard deviation.

Plasmid pTZ*sprP* was constructed harboring a transcriptional fusion of a 556 bp DNA fragment located upstream the *sprP* start codon with *lacZ* and transformed into *P. aeruginosa* PAO1. Bacteria were grown in LB medium and *β*-galactosidase activity was determined (Fig. 3). Activity of *β*-galactosidase steadily increased until it reached 1650 Miller units after 9 h to 12 h of growth at a cell density corresponding to an OD_580nm_ of 2.5. During the period from 9 h to 14 h, *β*-galactosidase activity remained constant and then started to decrease after 14 h. Apparently, the highest expression level of *sprP* is reached at an optical density of OD_580nm_ = 2.5; therefore, all further experiments were carried out with cells harvested at this optical density.

### Deletion of sprP promotes formation of cell aggregates and biofilms but abolishes cell motility

**Figure 4:**
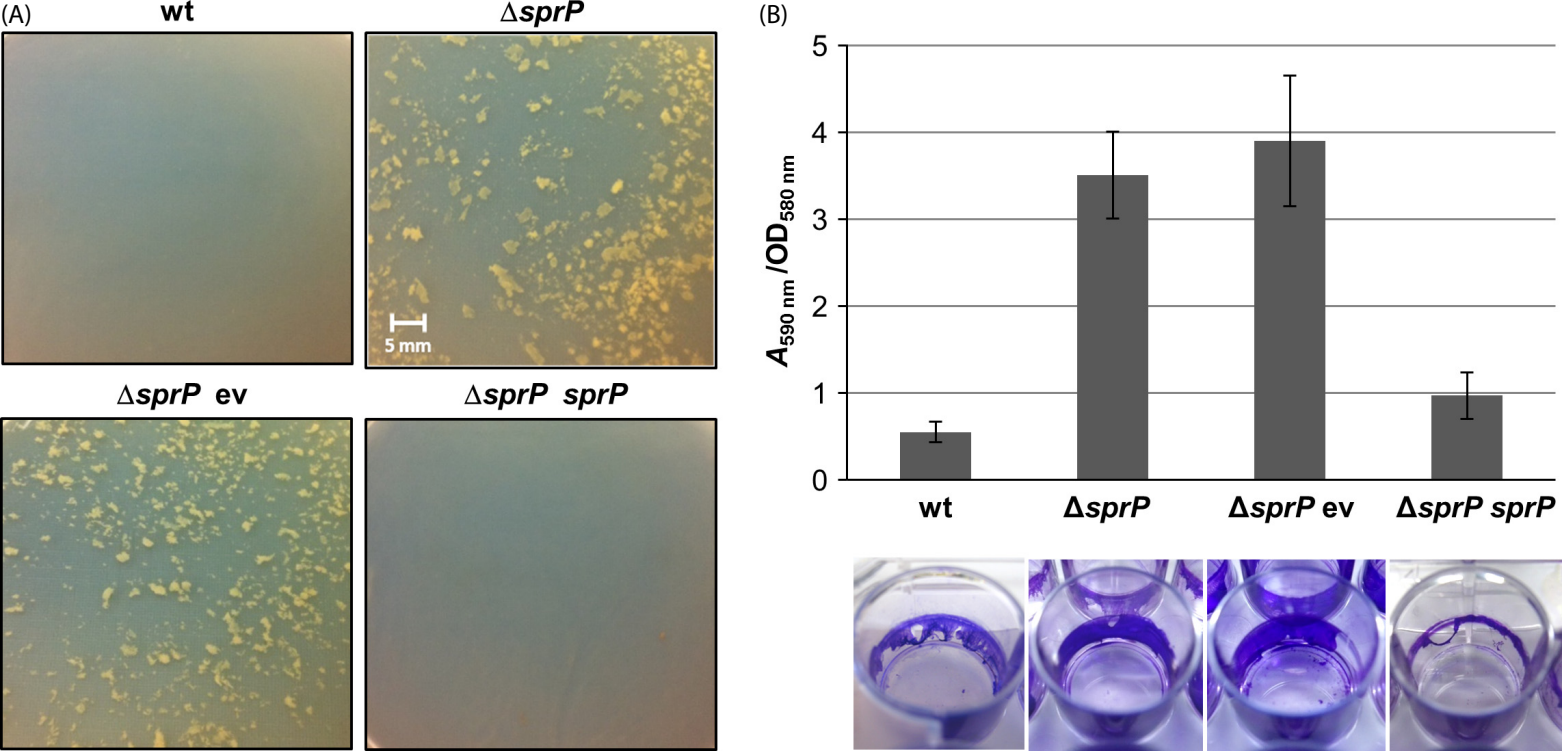
SprP affects cell aggregation and biofilm formation. *Pseudomonas aeruginosa* wild-type, Δ*sprP* mutant and Δ*sprP* mutant complemented with plasmid pBBRSP were inoculated to an initial cell density of OD_580nm_ = 0.1 and incubated until the cultures reached an OD_580nm_ of 2.5. (A) Cell aggregation was examined, cells transferred to petri dishes, and photographed. (B) Biofilm formation was analyzed in microtiter-plates inoculated with different strains and cultivated for 16 h at 37°C. Biofilms were stained with crystal violet, photographed, and dye was extracted with 100% ethanol and quantified photometrically at 590 nm. Biofilm formation is indicated as bound dye (*A*_590nm_) per cell density (OD_580nm_). Error bars indicate standard deviation. wt = *P. aeruginosa* PAO1, Δ*sprP = P. aeruginosa* Δ*sprP* harboring either ev = empty vector or *sprP* = plasmid pBBRSP.

A *sprP*-negative *P. aeruginosa* mutant was constructed to elucidate the physiological role of SprP. Upon growth of *P. aeruginosa ΔsprP*, extensive cell aggregation was observed during the entire cultivation period (Fig. 4A). Apparently, cell aggregation reached a maximum at maximal *sprP* expression; furthermore, aggregation of *P. aeruginosa* Δ*sprP* could be prevented by providing *sprP* on plasmid pBBRSP. Cell aggregation was shown to be linked to biofilm formation (Schleheck et al. 2009), we therefore analyzed biofilm formation using polystyrene microtiter-plates. *P. aeruginosa* Δ*sprP* showed an almost sixfold increased biofilm formation as compared to the wild-type strain *P. aeruginosa* PAO1 (Fig. 4B). Biofilm formation could also be reduced to almost wild-type level by complementation *in trans* of *P. aeruginosa* Δ*sprP* with *sprP*.

**Figure 5:**
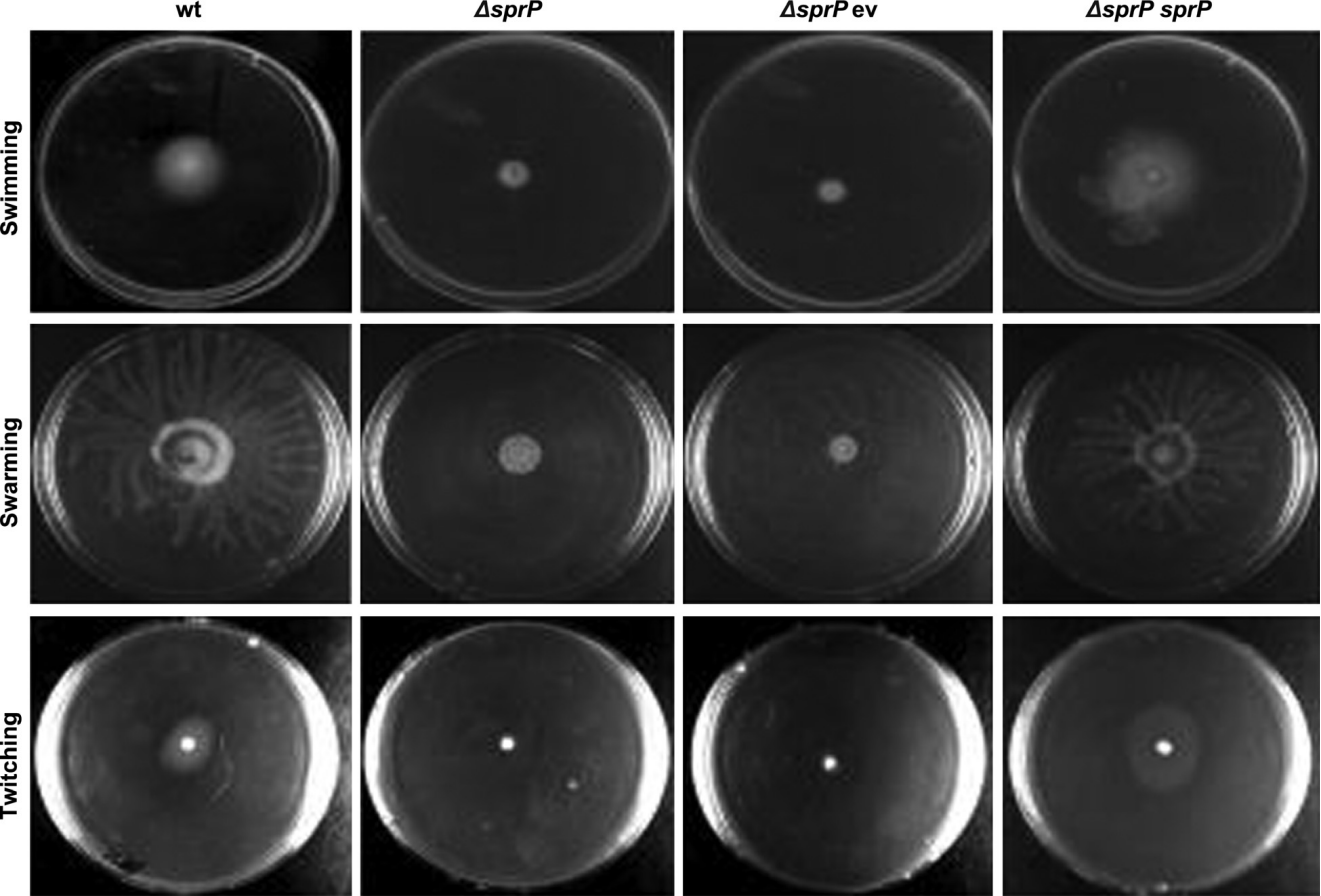
Deletion of *sprP* abolishes cell motility of *Pseudomonas aeruginosa*. Swarming and swimming agar plates contained M9 minimal medium with 0.5% agar for swarming and 0.3% agar for swimming. 5 *μ*L of bacterial culture at OD_580nm_ = 3 were added. Twitching motility was assessed after stabbing cells through 3 mm-thick LB agar plates onto the ground of the petri dishes. All plates were incubated for 16 h at 37°C. wt = *P. aeruginosa* PAO1, Δ*sprP = P. aeruginosa* Δ*sprP* harboring either ev = empty vector or *sprP* = plasmid pBBRSP.

Furthermore, biofilm formation by *P. aeruginosa* is linked to cell motility (Wilhelm et al. 2007; Rosenau et al. 2010; Tielen et al. 2010). Examination of *P. aeruginosa* Δ*sprP* for its swimming, swarming, and twitching motility revealed that all three types of motility were virtually abolished, but they could be reconstituted by complementation with plasmid-encoded *sprP* (Fig. 5).

### Deletion of sprP results in pleiotropic effects at the level of transcription

The transcriptomes of *P. aeruginosa* wild-type and Δ*sprP* mutant were comparatively analyzed (Tables S1 and S2). The strains were grown in LB medium until cell growth reached an OD_580nm_ of 2.5. A microarray analysis revealed the downregulation of 102 genes (Table S1) and upregulation of 116 genes (Table S2) in *P. aeruginosa* Δ*sprP*. Conditions were set such that regulation is ≥ twofold, *P*-value is ≤0.05, and the respective gene shows regulation in ≥3 independent experiments. Among the downregulated genes, 36 encode hypothetical proteins, five probable transcriptional regulators, and several genes are involved in the denitrification system. Upregulated genes include 46 encoding hypothetical proteins, three encoding probable transcriptional regulators, three sigma factors, and several genes belonging to the iron uptake system. A subset of the genes identified by transcriptome analysis was also tested for differential gene expression by quantitative PCR. The results confirmed the transcriptome data (Table S3).

### Deletion of sprP increases pyoverdine production and reduces growth under anaerobic conditions

**Figure 6:**
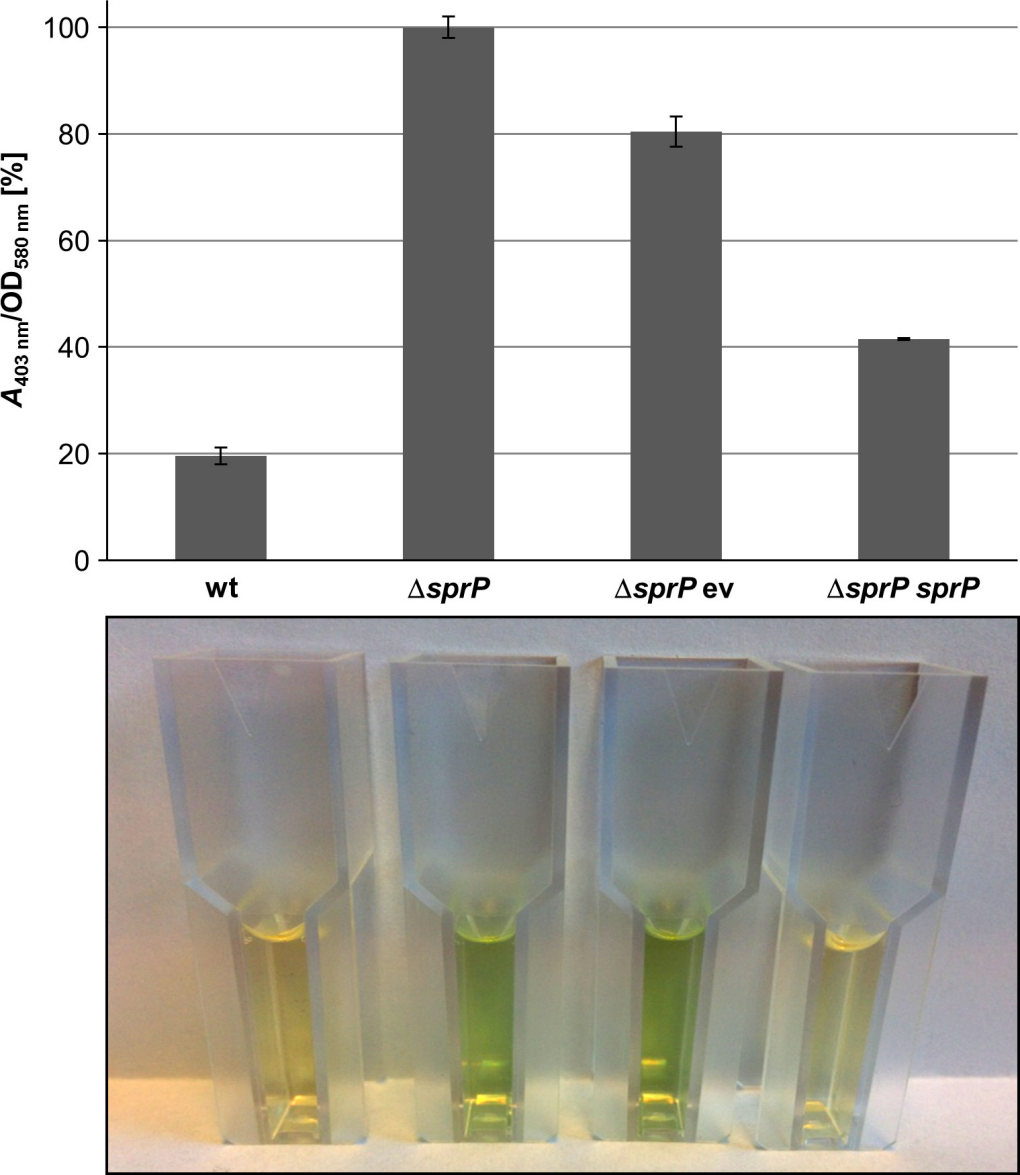
Deletion of *sprP* increases pyoverdine production by *Pseudomonas aeruginosa*. Strains were incubated at 37°C until cell growth reached an OD_580nm_ of 2.5 and pyoverdine in cell-free culture supernatants was determined by absorbance at 403 nm (Meyer and Abdallah 1978). Pyoverdine production is indicated as pyoverdine absorbance (*A*_403nm_) per cell density (OD_580 nm_). Error bars indicate standard deviation calculated from biological triplicates. Cuvettes containing culture supernatants are shown underneath the diagram. wt = *P. aeruginosa* PAO1, Δ*sprP = P. aeruginosa* Δ*sprP* harboring either ev = empty vector or *sprP* = plasmid pBBRSP.

In *P. aeruginosa* Δ*sprP* several genes involved in pyoverdine production were strongly upregulated. These results are consistent with the observation that a *P. aeruginosa* Δ*sprP* culture showed an intense green color indicating strongly increased production of pyoverdines. The culture supernatant of *P. aeruginosa ΔsprP* contained almost fivefold the amount of pyoverdine as compared to the wild-type supernatant, complementation with the *sprP* gene reduced pyoverdine concentration to about wild-type level (Fig. 6).

**Figure 7:**
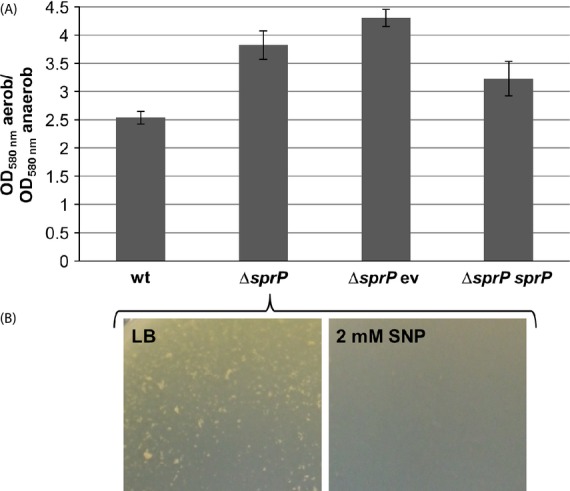
SprP affects anaerobic growth of *Pseudomonas aeruginosa*. (A) Bacterial cultures were incubated at 37°C for 6 h and growth differences under anaerobic and aerobic conditions are illustrated. (B) The cell aggregation phenotype of *P. aeruginosa* Δ*sprP* after cultivation in LB-medium and LB-medium supplemented with the nitric oxide donor sodium nitroprusside (SNP). wt = *P. aeruginosa* PAO1, Δ*sprP = P. aeruginosa* Δ*sprP* harboring either ev = empty vector or *sprP* = plasmid pBBRSP.

Additionally, we have analyzed growth of *P. aeruginosa* wild-type and Δ*sprP* under anaerobic conditions (Fig. 7A) by incubation at 37°C with rotary shaking after 3, 6, and 8 h. The most striking difference in cell growth was observed after 6 h when the cell density was 2.5 times lower under anaerobic than under aerobic conditions for the wild type and 3.8-fold lower for the Δ*sprP* mutant. The deficiency in anaerobic growth can largely be reverted by expression of the *sprP* gene in *P. aeruginosa* Δ*sprP*. Transcriptome analysis had shown that deletion of *sprP* lead to a downregulation of genes involved in the denitrification system. Hence, we assumed that the level of the denitrification product nitric oxide (NO) may be reduced. Thus, we added the synthetic NO donor SNP to the growth medium and cultivated the strains until a cell density of OD_580nm_ = 2.5 was reached. Interestingly, addition of 2 mmol/L SNP to the growth medium largely abrogated the cell aggregation phenotype of *P. aeruginosa* Δ*sprP* (Fig. 7B).

## Discussion

Proteases are important bacterial virulence factors and several of them are well known for their interaction with host cells during the infection process (Hoge et al. 2010; Pearson et al. 2011; Kida et al. 2013; Tang et al. 2013). The MEROPS peptidase database lists 284 known and putative proteases and 135 non-peptidase homologues for *P. aeruginosa* (Rawlings et al. 2012). We have newly identified the subtilase SprP of *P. aeruginosa* which consists of three distinct domains. The large N-terminal (DUF, 233 aa) shows high homology to several hypothetical proteins and putative subtilases. Remarkably, these homologous proteins are almost exclusively encoded in genomes of bacteria belonging to the genus *Pseudomonas* suggesting a *Pseudomonas*-specific function. Deletion of domains DUF and S8 both lead to the loss of protease activity of SprP. Also, both constructs were unable to complement the phenotypes observed for *P. aeruginosa* Δ*sprP* (data not shown) indicating that full-length SprP is required. Most bacterial subtilases possess aspartic acid, histidine, and serine as catalytic residues thus belonging to the large group of D-H-S family subtilases. For SprP, glutamic acid, histidine, and serine were predicted as catalytic residues thereby assigning SprP to the E-H-S family of subtilases based on the Prokaryotic Subtilase Database (Siezen et al. 2007). SprP variant S499A which had the catalytic serine replaced by alanine did not show protease activity thus corroborating this assumption.

The predicted protease function of PA1242 was demonstrated after expression in *E. coli* on skim milk agar plates and by using the subtilisin-specific substrate Suc-AAPF-*p*NA (Brode et al. 1996) (Fig. 2). Experiments using a *sprP*::*lacZ* fusion demonstrated maximal expression of *sprP* at the onset of the stationary growth phase (Fig. 3). However, the absolute promoter activity (indicated in Miller units) was comparatively low (Morabbi Heravi et al. 2011; Salto et al. 1998) suggesting a low expression level of *sprP* in *P. aeruginosa*.

Transcriptome analyses revealed the highest level of upregulation for the genes *antA*BC (max. 44.8-fold) and *catBCA* (max 20-fold) (Table S2). Both operons are involved in the degradation of anthranilate into TCA cycle intermediates (Chang et al. 2003; Urata et al. 2004; Choi et al. 2011). *antA* is directly activated by the activator AntR (Oglesby et al. 2008; Kim et al. 2012), which is shown to be upregulated after *sprP* deletion as well (3.9-fold) (Table S2). We did not analyze these effects in more detail but this study indicates that SprP could be of interest for the identification of regulators for the partly unknown regulation of the anthranilate metabolism (Choi et al. 2011). A *P. aeruginosa* Δ*sprP* mutant showed strong cell aggregation throughout the entire growth period (Fig. 4A). We also observed that these cell aggregates attach to a polystyrene surface resulting in significantly increased biofilm formation (Fig. 4B). Previously, it was shown that *P. aeruginosa* PAO1 cells also form aggregates in batch cultures (Schleheck et al. 2009). These aggregates are comparatively small with up to 600 *μ*m in relation to the aggregates formed by the Δ*sprP* mutant. Furthermore, it was demonstrated that the expression of *cdrAB* genes resulted in increased biofilm formation and auto-aggregation in liquid cultures (Borlee et al. 2010). Interestingly, both genes are also upregulated in the *P. aeruginosa* Δ*sprP* strain (*cdrA* 3.3-fold, *cdrB* 4.1-fold; Table S2). Proteins encoded by the *cdrAB* operon regulate the intracellular level of c-di-GMP in *P. aeruginosa* that is linked to the production of matrix components and biofilm formation (Tischler and Camilli 2004; Hickman et al. 2005; Borlee et al. 2010; Povolotsky and Hengge 2012). The loss of motility observed for *P. aeruginosa* Δ*sprP* may represent another reason for cellular aggregation and increased biofilm formation. Transcriptome analyses of *P. aeruginosa* Δ*sprP* revealed a downregulation of *pilA* gene (2.5-fold) expression (Table S1). Previous studies have demonstrated that a *pilA* mutant showed a non-twitching phenotype (Chiang and Burrows 2003; Shrout et al. 2006) and may also be defective for swarming (Köhler et al. 2000).

Another surprising observation resulting from transcriptome analysis of *P. aeruginosa* Δ*sprP* refers to downregulation of the genes *narK1* (21.3-fold), *narK2* (5.9-fold), *narJ* (5.8-fold), *narH* (3.5-fold), *narG* (7.2-fold), *nirN* (2.8-fold), *nirJ* (2.1-fold), *nirL* (2.5-fold), *nirF* (3.9-fold), *nirS* (fivefold), *nosZ* (14.5-fold), *nosD* (4.5-fold) (Table S1), which are organized in five operons (Schobert and Jahn 2010) and are involved in denitrification and anaerobic growth of *P. aeruginosa*. Indeed, we observed a significantly impaired growth of *P. aeruginosa ΔsprP* under anaerobic conditions as compared to the wild-type strain (Fig. 7A). NO, a side product of denitrification, can induce dispersal of *P. aeruginosa* biofilms (Barraud et al. 2006, 2009). We found that genes encoding nitrate reductase (NarGHI) and nitrite reductase (NirS), which are involved in NO production were downregulated in *P. aeruginosa* Δ*sprP*. Thus, we speculated that the level of NO might be reduced in the mutant. As expected, addition of nitroprusside as an alternative source of NO significantly reduced cell aggregation of *P. aeruginosa* Δ*sprP* (Fig. 7B). Another identified probable protease PA3913 was already linked to anaerobic growth of *P. aeruginosa* (Filiatrault et al. 2006). It was shown that transposon mutants of PA3913 are unable to grow anaerobically for unknown reasons. This study shows that the putative protease PA3913 is also downregulated 6.4-fold in *P. aeruginosa* Δ*sprP* (Table S1). Presumably, both SprP and PA3913 are involved in regulation of anaerobic growth in *P. aeruginosa*.

The pyoverdine system is well characterized in *P. aeruginosa* (Ochsner et al. 2002; Lamont and Martin 2003; Cornelis 2010; Funken et al. 2011). We found significant upregulation of expression levels of regulatory genes *fpvA* (2.4-fold), *fpvR* (twofold), and *pvdS* (2.1-fold) as well as genes *pvdA* (sevenfold), *pvdN* (fivefold), *pvdO* (4.3-fold), *pvdL* (2.8-fold), and *pvdH* (2.1-fold) (Table S2) resulting in significantly increased pyoverdine production by *P. aeruginosa* Δ*sprP*. Increased pyoverdine synthesis and formation of cell aggregates were also observed when *P. aeruginosa* was grown under iron-limitation in M9 minimal media with succinate as sole carbon source (data not shown).

The relevance of proteases as part of regulatory networks and for virulence of *P. aeruginosa* was recently demonstrated (Breidenstein et al. 2012; Fernandez et al. 2012). Apparently, several intracellular proteases are involved in the regulation of different phenotypes like biofilm formation, motility, and antibiotic resistance. The exact cellular location of SprP is presently unknown, but an N-terminal signal sequence was predicted and results of Blonder et al. 2004 indicated that SprP is located in the membrane fraction isolated from *P. aeruginosa* suggesting that SprP may be located in the periplasm associated with the cytoplasmic or the outer membrane. This study adds protease SprP to the collection of *P. aeruginosa* proteases with an important regulatory function. As a next step, intracellular substrates for SprP need to be identified, for example, by isotope labeling and subsequent mass spectroscopic analyses.

## Supporting Information

**Table S1.** Selected genes downregulated in *Pseudomonas aeruginosa ΔsprP* in comparison to the wild-type strain. **Table S2.** Selected genes upregulated in *Pseudomonas aeruginosa ΔsprP* in comparison to the wild-type strain. **Table S3.** Validation of selected genes from microarray analysis by qPCR. **Table S4.** Primer used for qPCR in this study.
