# Supplementary material for: Subtilase SprP exerts pleiotropic effects in Pseudomonas aeruginosa
Source: Microbiologyopen. 2013 Dec 26;3(1):89–103. doi: 10.1002/mbo3.150 (PMC3937732; doi:10.1002/mbo3.150)
Supplement: Supplementary file 1 — Table S1. Selected genes downregulated in Pseudomonas aeruginosa ΔsprP in comparison to the wild-type strain. Table S2. Selected genes upregulated in Pseudomonas aeruginosa ΔsprP in comparison to the wild-type strain. Table S3. Validation of selected genes from microarray analysis by qPCR. Table S4. Primer used for qPCR in this study. [file mbo30003-0089-sd1.docx]

**Supplementary Data**

**Table S1.** Selected genes down-regulated in *P. aeruginosa* ∆*sprP* in comparison to the wild-type strain. The chosen criteria are a fold change of ≥ 2, a p-value ≤ 0.05, and the average illustrates the mean of the relative mRNA level wild-type / *P. aeruginosa* Δ*sprP* of at least 3 biological replicate experiments. Adjusted p-value gives the value adjusted to the Hochberg method. * Designation from the *Pseudomonas* Genome Database (www.pseudomonas.com).

| **Name** | **Symbol*** | **Average** | **p-value** | **adjusted p-value** | **Annotation** |
| --- | --- | --- | --- | --- | --- |
| PA0048 | - | 3,5992 | 0,04304 | 0,19999 | probable transcriptional regulator |
| PA0049 | - | 4,5620 | 0,04336 | 0,19239 | hypothetical protein |
| PA0050 | - | 2,8795 | 0,04090 | 0,20280 | hypothetical protein |
| PA0070 | - | 2,3664 | 0,00733 | 0,06473 | TagQ1 |
| PA0085 | hcp1 | 2,4499 | 0,03082 | 0,16742 | Hcp1 |
| PA0130 | - | 2,3864 | 0,03393 | 0,20054 | 3-Oxopropanoate dehydrogenase |
| PA0131 | - | 2,1433 | 0,03217 | 0,19178 | hypothetical protein |
| PA0132 | - | 2,2620 | 0,02698 | 0,16798 | Beta-alanine:pyruvate transaminase |
| PA0176 | aer2 | 2,4339 | 0,00131 | 0,02298 | aerotaxis transducer Aer2 |
| PA0210 | mdcC | 2,1090 | 0,02076 | 0,14774 | malonate decarboxylase delta subunit |
| PA0263 | hcpC | 4,3707 | 0,00237 | 0,01732 | secreted protein Hcp |
| PA0291 | oprE | 3,0186 | 0,00944 | 0,06422 | Anaerobically-induced outer membrane porin OprE precursor |
| PA0320 | - | 5,1426 | 0,02514 | 0,29001 | conserved hypothetical protein |
| PA0509 | nirN | 2,8314 | 0,04596 | 0,24095 | probable c-type cytochrome |
| PA0510 | - | 2,4117 | 0,02964 | 0,16760 | probable uroporphyrin-III c-methyltransferase |
| PA0511 | nirJ | 2,1097 | 0,03872 | 0,22112 | heme d1 biosynthesis protein NirJ |
| PA0512 | - | 3,3982 | 0,04780 | 0,24553 | conserved hypothetical protein |
| PA0513 | - | 3,2369 | 0,02486 | 0,13627 | probable transcriptional regulator |
| PA0514 | nirL | 2,4480 | 0,03802 | 0,20546 | heme d1 biosynthesis protein NirL |
| PA0515 | - | 3,2243 | 0,03723 | 0,18263 | probable transcriptional regulator |
| PA0516 | nirF | 3,9214 | 0,03066 | 0,14933 | heme d1 biosynthesis protein NirF |
| PA0519 | nirS | 4,9993 | 0,04969 | 0,20829 | nitrite reductase precursor |
| PA0526 | - | 3,9184 | 0,01911 | 0,10269 | hypothetical protein |
| PA0754 | - | 2,9749 | 0,01305 | 0,07732 | hypothetical protein |
| PA0755 | opdH | 3,1859 | 0,00498 | 0,03807 | cis-aconitate porin OpdH |
| PA0796 | prpB | 2,0139 | 0,00347 | 0,09898 | carboxyphosphonoenolpyruvate phosphonomutase |
| PA1070 | braG | 2,0915 | 0,01357 | 0,11926 | branched-chain amino acid transport protein BraG |
| PA1511 | - | 2,0971 | 0,00444 | 0,06064 | conserved hypothetical protein |
| PA1512 | hcpA | 4,0780 | 0,00269 | 0,02036 | secreted protein Hcp |
| PA1609 | fabB | 2,0043 | 0,00658 | 0,08032 | beta-ketoacyl-ACP synthase I |
| PA1656 | - | 2,4142 | 0,00322 | 0,04282 | hypothetical protein |
| PA1657 | - | 3,2754 | 0,00462 | 0,03395 | conserved hypothetical protein |
| PA1658 | - | 3,0293 | 0,00390 | 0,03476 | conserved hypothetical protein |
| PA1659 | - | 2,2642 | 0,00080 | 0,02765 | hypothetical protein |
| PA1661 | - | 2,2365 | 0,01764 | 0,07909 | hypothetical protein |
| PA1662 | - | 2,3726 | 0,00618 | 0,08935 | probable ClpA/B-type protease |
| PA1663 | - | 2,0679 | 0,01732 | 0,13972 | probable transcriptional regulator |
| PA1666 | - | 2,1575 | 0,01858 | 0,13438 | hypothetical protein |
| PA1668 | - | 2,3555 | 0,04536 | 0,06549 | hypothetical protein |
| PA1669 | - | 2,3130 | 0,00395 | 0,04580 | hypothetical protein |
| PA1670 | stp1 | 2,3713 | 0,03606 | 0,11779 | serine/threonine phosphoprotein phosphatase Stp1 |
| PA1846 | cti | 2,0135 | 0,00540 | 0,05563 | cis/trans isomerase |
| PA1913 | - | 2,8938 | 0,01434 | 0,01961 | hypothetical protein |
| PA1914 | - | 2,4323 | 0,00570 | 0,06855 | conserved hypothetical protein |
| PA1984 | - | 2,0594 | 0,02001 | 0,14471 | NAD+ dependent aldehyde dehydrogenase ExaC |
| PA2012 | liuD | 2,4062 | 0,00790 | 0,06246 | methylcrotonyl-CoA carboxylase, alpha-subunit (biotin-containing) |
| PA2013 | liuC | 2,0342 | 0,00133 | 0,05090 | putative 3-methylglutaconyl-CoA hydratase |
| PA2014 | liuB | 2,3824 | 0,00194 | 0,03335 | methylcrotonyl-CoA carboxylase, beta-subunit |
| PA2110 | - | 4,0248 | 0,03053 | 0,14976 | hypothetical protein |
| PA2111 | - | 3,6203 | 0,03279 | 0,15453 | hypothetical protein |
| PA2114 | - | 3,3808 | 0,04853 | 0,21567 | probable major facilitator superfamily (MFS) transporter |
| PA2317 | - | 3,4483 | 0,04030 | 0,18855 | probable oxidoreductase |
| PA2318 | - | 3,7397 | 0,04454 | 0,19664 | hypothetical protein |
| PA2552 | - | 2,0028 | 0,01216 | 0,11398 | probable acyl-CoA dehydrogenase |
| PA2729 | - | 2,3801 | 0,01096 | 0,04201 | hypothetical protein |
| PA3328 | - | 2,6119 | 0,00326 | 0,03500 | probable FAD-dependent monooxygenase |
| PA3329 | - | 2,2304 | 0,00490 | 0,05639 | hypothetical protein |
| PA3330 | - | 2,5312 | 0,00125 | 0,02723 | probable short chain dehydrogenase |
| PA3332 | - | 2,2534 | 0,00363 | 0,04763 | conserved hypothetical protein |
| PA3333 | fabH2 | 2,0148 | 0,00203 | 0,06542 | 3-oxoacyl-[acyl-carrier-protein] synthase III |
| PA3334 | - | 2,4404 | 0,00681 | 0,06181 | probable acyl carrier protein |
| PA3335 | - | 2,7617 | 0,00945 | 0,06900 | hypothetical protein |
| PA3392 | nosZ | 14,5280 | 0,01048 | 0,12944 | nitrous-oxide reductase precursor |
| PA3393 | nosD | 4,4900 | 0,00944 | 0,08188 | NosD protein |
| PA3686 | adk | 2,1544 | 0,01444 | 0,11733 | adenylate kinase |
| PA3762 | - | 2,4755 | 0,02605 | 0,16437 | hypothetical protein |
| PA3820 | secF | 2,0350 | 0,01826 | 0,12064 | secretion protein SecF |
| PA3873 | narJ | 5,8144 | 0,04219 | 0,21917 | respiratory nitrate reductase delta chain |
| PA3874 | narH | 3,5102 | 0,01058 | 0,05731 | respiratory nitrate reductase beta chain |
| PA3875 | narG | 7,1694 | 0,00771 | 0,04120 | respiratory nitrate reductase alpha chain |
| PA3876 | narK2 | 5,9346 | 0,00142 | 0,01022 | nitrite extrusion protein 2 |
| PA3877 | narK1 | 21,3321 | 0,00132 | 0,00575 | nitrite extrusion protein 1 |
| PA3905 | - | 2,0433 | 0,00022 | 0,03033 | hypothetical protein |
| PA3906 | - | 2,0203 | 0,00064 | 0,03973 | hypothetical protein |
| PA3907 | - | 2,0451 | 0,00394 | 0,07030 | hypothetical protein |
| PA3908 | - | 2,0516 | 0,00000 | 0,03234 | hypothetical protein |
| PA3912 | - | 5,7430 | 0,00724 | 0,03005 | conserved hypothetical protein |
| PA3913 | - | 6,4355 | 0,00533 | 0,03310 | probable protease |
| PA3914 | moeA1 | 3,5246 | 0,02108 | 0,04616 | molybdenum cofactor biosynthetic protein A1 |
| PA3915 | moaB1 | 8,2757 | 0,00407 | 0,02238 | molybdopterin biosynthetic protein B1 |
| PA3916 | moaE | 2,6050 | 0,02017 | 0,12573 | molybdopterin converting factor, large subunit |
| PA3917 | moaD | 2,3534 | 0,02494 | 0,15282 | molybdopterin converting factor, small subunit |
| PA3918 | moaC | 2,6379 | 0,04793 | 0,23006 | molybdopterin biosynthetic protein C |
| PA4022 | - | 2,0602 | 0,01953 | 0,14326 | hydrazone dehydrogenase, HdhA |
| PA4063 | - | 2,0733 | 0,00761 | 0,08773 | hypothetical protein |
| PA4152 | - | 3,1411 | 0,01129 | 0,07230 | probable hydrolase |
| PA4153 | - | 3,6127 | 0,03054 | 0,09342 | 2,3-butanediol dehydrogenase |
| PA4294 | - | 2,2902 | 0,00443 | 0,05398 | hypothetical protein |
| PA4333 | - | 2,7089 | 0,03083 | 0,23078 | probable fumarase |
| PA4489 | - | 2,3571 | 0,00388 | 0,04625 | conserved hypothetical protein |
| PA4525 | pilA | 2,5316 | 0,01237 | 0,08322 | type 4 fimbrial precursor PilA |
| PA4659 | - | 3,1361 | 0,02648 | 0,13956 | probable transcriptional regulator |
| PA4683 | - | 2,4798 | 0,00261 | 0,03806 | hypothetical protein |
| PA4888 | desB | 2,4491 | 0,00637 | 0,06205 | acyl-CoA delta-9-desaturase, DesB |
| PA5266 | - | 4,2786 | 0,01225 | 0,02402 | conserved hypothetical protein |
| PA5267 | hcpB | 3,8789 | 0,00345 | 0,02949 | secreted protein Hcp |
| PA5360 | phoB | 2,1367 | 0,03049 | 0,18435 | two-component response regulator PhoB |
| PA5368 | pstC | 2,2598 | 0,04323 | 0,22591 | membrane protein component of ABC phosphate transporter |
| PA5435 | - | 6,4758 | 0,00665 | 0,03603 | probable transcarboxylase subunit |
| PA5436 | - | 3,6542 | 0,00241 | 0,02009 | probable biotin carboxylase subunit of a transcarboxylase |
| PA5479 | gltP | 2,5281 | 0,00130 | 0,03595 | proton-glutamate symporter |
| PA5549 | glmS | 2,4143 | 0,00015 | 0,01667 | glucosamine--fructose-6-phosphate aminotransferase |

**Table S2.** Selected genes up-regulated in *P. aeruginosa* ∆*sprP* in comparison to the wild-type strain. The chosen criteria are a fold change of ≥ 2, a p-value ≤ 0.05, and the average illustrates the mean of the relative mRNA level wild-type / *P. aeruginosa* Δ*sprP* of at least 3 biological replicate experiments. Adjusted p-value gives the value adjusted to the Hochberg method. * Designation from the *Pseudomonas* Genome Database (www.pseudomonas.com).

| **Name** | **Symbol*** | **Average** | **p-value** | **adjusted**  **p-value** | **Annotation** |
| --- | --- | --- | --- | --- | --- |
| PA0169 | - | 2,0704 | 0,00024 | 0,05189 | SiaD |
| PA0170 | - | 2,9301 | 0,01204 | 0,07040 | hypothetical protein |
| PA0171 | - | 2,6531 | 0,00999 | 0,07143 | hypothetical protein |
| PA0227 | - | 3,2139 | 0,04792 | 0,18034 | probable CoA transferase, subunit B |
| PA0228 | pcaF | 3,2707 | 0,01531 | 0,06658 | beta-ketoadipyl CoA thiolase PcaF |
| PA0229 | pcaT | 3,3795 | 0,03076 | 0,05338 | dicarboxylic acid transporter PcaT |
| PA0366 | - | 2,1239 | 0,00940 | 0,07905 | probable aldehyde dehydrogenase |
| PA0534 | - | 4,7311 | 0,00775 | 0,04274 | FAD-dependent oxidoreductase |
| PA0535 | - | 3,1183 | 0,00569 | 0,03749 | probable transcriptional regulator |
| PA0613 | - | 2,2967 | 0,04178 | 0,20097 | hypothetical protein |
| PA0719 | - | 2,1484 | 0,00215 | 0,16702 | hypothetical protein of bacteriophage Pf1 |
| PA0839 | - | 2,9328 | 0,03613 | 0,16780 | probable transcriptional regulator |
| PA1168 | - | 31,3233 | 0,00009 | 0,00047 | hypothetical protein |
| PA1169 | - | 19,7484 | 0,00027 | 0,00192 | probable lipoxygenase |
| PA1219 | - | 2,0107 | 0,01522 | 0,10874 | hypothetical protein |
| PA1317 | cyoA | 9,5242 | 0,00005 | 0,00155 | cytochrome o ubiquinol oxidase subunit II |
| PA1318 | cyoB | 7,6256 | 0,00012 | 0,00203 | cytochrome o ubiquinol oxidase subunit I |
| PA1319 | cyoC | 4,0353 | 0,04107 | 0,05357 | cytochrome o ubiquinol oxidase subunit III |
| PA1351 | - | 2,1125 | 0,00104 | 0,12524 | probable sigma-70 factor, ECF subfamily |
| PA1596 | htpG | 2,7184 | 0,00013 | 0,00875 | heat shock protein HtpG |
| PA1784 | - | 2,0066 | 0,02391 | 0,16813 | hypothetical protein |
| PA1888 | - | 2,0183 | 0,02930 | 0,16366 | hypothetical protein |
| PA1927 | metE | 3,2042 | 0,00976 | 0,15238 | 5-methyltetrahydropteroyltriglutamate-homocysteine S-methyltransferase |
| PA2031 | - | 2,1690 | 0,00356 | 0,05199 | hypothetical protein |
| PA2068 | - | 2,2037 | 0,00638 | 0,06636 | probable major facilitator superfamily (MFS) transporter |
| PA2137 | - | 2,0565 | 0,04549 | 0,04573 | hypothetical protein |
| PA2146 | - | 3,3289 | 0,00067 | 0,00777 | conserved hypothetical protein |
| PA2156 | - | 2,4249 | 0,00554 | 0,14969 | conserved hypothetical protein |
| PA2158 | - | 2,3191 | 0,02804 | 0,12836 | probable alcohol dehydrogenase (Zn-dependent) |
| PA2159 | - | 2,4263 | 0,00056 | 0,01747 | conserved hypothetical protein |
| PA2160 | - | 2,4282 | 0,00719 | 0,05815 | probable glycosyl hydrolase |
| PA2161 | - | 2,3813 | 0,01537 | 0,09900 | hypothetical protein |
| PA2162 | - | 2,1525 | 0,00139 | 0,04289 | probable glycosyl hydrolase |
| PA2163 | - | 2,0680 | 0,00098 | 0,04238 | hypothetical protein |
| PA2164 | - | 2,3000 | 0,01712 | 0,07705 | probable glycosyl hydrolase |
| PA2169 | - | 2,9999 | 0,00548 | 0,03151 | hypothetical protein |
| PA2171 | - | 2,2867 | 0,00056 | 0,02095 | hypothetical protein |
| PA2190 | - | 2,2580 | 0,01255 | 0,08214 | conserved hypothetical protein |
| PA2300 | chiC | 2,0339 | 0,00457 | 0,05645 | chitinase |
| PA2386 | pvdA | 7,0309 | 0,00054 | 0,00376 | L-ornithine N5-oxygenase |
| PA2388 | fpvR | 2,0199 | 0,00565 | 0,07598 | FpvR |
| PA2393 | - | 2,4556 | 0,01660 | 0,06103 | probable dipeptidase precursor |
| PA2394 | pvdN | 4,9841 | 0,01545 | 0,03911 | PvdN |
| PA2395 | pvdO | 4,2518 | 0,02684 | 0,05131 | PvdO |
| PA2398 | fpvA | 2,4401 | 0,00929 | 0,06263 | ferripyoverdine receptor |
| PA2403 | - | 2,0002 | 0,00669 | 0,07309 | hypothetical protein |
| PA2404 | - | 2,5288 | 0,01756 | 0,09795 | hypothetical protein |
| PA2405 | - | 2,2969 | 0,01403 | 0,09495 | hypothetical protein |
| PA2412 | - | 3,1155 | 0,02271 | 0,06353 | conserved hypothetical protein |
| PA2413 | pvdH | 2,0875 | 0,02482 | 0,08062 | L-2,4-diaminobutyrate:2-ketoglutarate 4-aminotransferase, PvdH |
| PA2414 | - | 2,0235 | 0,00092 | 0,04603 | L-sorbosone dehydrogenase |
| PA2424 | pvdL | 2,7731 | 0,01748 | 0,08437 | PvdL |
| PA2426 | pvdS | 2,0840 | 0,01652 | 0,11452 | sigma factor PvdS |
| PA2433 | - | 2,2282 | 0,00100 | 0,02466 | hypothetical protein |
| PA2440 | - | 2,5106 | 0,01687 | 0,04724 | hypothetical protein |
| PA2441 | - | 3,7900 | 0,00410 | 0,02729 | hypothetical protein |
| PA2504 | - | 2,1169 | 0,02840 | 0,15951 | hypothetical protein |
| PA2507 | catA | 16,8336 | 0,01870 | 0,04046 | catechol 1,2-dioxygenase |
| PA2508 | catC | 20,0445 | 0,00100 | 0,00440 | muconolactone delta-isomerase |
| PA2509 | catB | 9,9833 | 0,00616 | 0,01961 | muconate cycloisomerase I |
| PA2511 | - | 3,8857 | 0,00227 | 0,01763 | probable transcriptional regulator |
| PA2512 | antA | 44,7633 | 0,00041 | 0,00217 | anthranilate dioxygenase large subunit |
| PA2513 | antB | 32,8900 | 0,00036 | 0,00230 | anthranilate dioxygenase small subunit |
| PA2514 | antC | 27,5463 | 0,00056 | 0,00336 | anthranilate dioxygenase reductase |
| PA2682 | - | 6,7014 | 0,00188 | 0,00970 | conserved hypothetical protein |
| PA2700 | opdB | 2,0154 | 0,01677 | 0,07087 | proline porin OpdB |
| PA2927 | - | 2,1783 | 0,00817 | 0,08079 | hypothetical protein |
| PA3032 | snr1 | 3,0168 | 0,00022 | 0,00816 | cytochrome c Snr1 |
| PA3126 | ibpA | 2,1527 | 0,02935 | 0,16059 | heat-shock protein IbpA |
| PA3187 | - | 2,6494 | 0,01097 | 0,14279 | probable ATP-binding component of ABC transporter |
| PA3188 | - | 2,4712 | 0,03114 | 0,16073 | probable permease of ABC sugar transporter |
| PA3189 | - | 2,2627 | 0,02563 | 0,16460 | probable permease of ABC sugar transporter |
| PA3190 | - | 3,0122 | 0,00723 | 0,03506 | probable binding protein component of ABC sugar transporter |
| PA3369 | - | 2,3366 | 0,00456 | 0,04467 | hypothetical protein |
| PA3370 | - | 3,5092 | 0,00434 | 0,03139 | hypothetical protein |
| PA3371 | - | 3,2127 | 0,00012 | 0,00502 | hypothetical protein |
| PA3459 | - | 2,1649 | 0,00480 | 0,05641 | probable glutamine amidotransferase |
| PA3460 | - | 2,0988 | 0,02111 | 0,13740 | probable acetyltransferase |
| PA3478 | rhlB | 2,3231 | 0,01824 | 0,10492 | rhamnosyltransferase chain B |
| PA3479 | rhlA | 2,3195 | 0,00896 | 0,06360 | rhamnosyltransferase chain A |
| PA3622 | rpoS | 2,1164 | 0,00394 | 0,04195 | sigma factor RpoS |
| PA3815 | - | 2,6211 | 0,00562 | 0,04406 | IscR |
| PA3960 | - | 2,7521 | 0,01327 | 0,08261 | hypothetical protein |
| PA4078 | - | 2,6145 | 0,00008 | 0,00787 | probable nonribosomal peptide synthetase |
| PA4139 | - | 25,8568 | 0,00006 | 0,00037 | hypothetical protein |
| PA4140 | - | 13,1334 | 0,00306 | 0,01443 | hypothetical protein |
| PA4141 | - | 2,2996 | 0,01067 | 0,08242 | hypothetical protein |
| PA4142 | - | 2,5383 | 0,00001 | 0,00766 | probable secretion protein |
| PA4205 | mexG | 2,7909 | 0,00548 | 0,06674 | hypothetical protein |
| PA4206 | mexH | 3,8180 | 0,00393 | 0,02636 | probable Resistance-Nodulation-Cell Division (RND) efflux membrane fusion protein precursor |
| PA4207 | mexI | 2,2111 | 0,00629 | 0,06195 | probable Resistance-Nodulation-Cell Division (RND) efflux transporter |
| PA4223 | - | 2,1952 | 0,04484 | 0,21016 | probable ATP-binding component of ABC transporter |
| PA4225 | pchF | 2,1957 | 0,02994 | 0,15510 | pyochelin synthetase |
| PA4226 | pchE | 2,3325 | 0,04768 | 0,21331 | dihydroaeruginoic acid synthetase |
| PA4228 | pchD | 4,2199 | 0,00010 | 0,00336 | pyochelin biosynthesis protein PchD |
| PA4229 | pchC | 3,0350 | 0,00537 | 0,04297 | pyochelin biosynthetic protein PchC |
| PA4230 | pchB | 3,6177 | 0,00542 | 0,03656 | salicylate biosynthesis protein PchB |
| PA4359 | feoA | 2,5674 | 0,00296 | 0,03355 | conserved hypothetical protein |
| PA4377 | - | 2,0414 | 0,00025 | 0,02724 | hypothetical protein |
| PA4468 | sodM | 4,5990 | 0,00510 | 0,02964 | superoxide dismutase |
| PA4469 | - | 5,7207 | 0,00299 | 0,02444 | hypothetical protein |
| PA4570 | - | 2,6241 | 0,00312 | 0,05431 | hypothetical protein |
| PA4624 | - | 4,0599 | 0,01415 | 0,06820 | cyclic diguanylate-regulated TPS partner B, CdrB |
| PA4625 | - | 3,2725 | 0,02314 | 0,10858 | cyclic diguanylate-regulated TPS partner A, CdrA |
| PA4709 | - | 2,0447 | 0,04506 | 0,06752 | probable hemin degrading factor |
| PA4739 | - | 2,5473 | 0,01359 | 0,07578 | conserved hypothetical protein |
| PA5056 | phaC1 | 2,3546 | 0,01567 | 0,10142 | poly(3-hydroxyalkanoic acid) synthase 1 |
| PA5058 | phaC2 | 2,3275 | 0,01780 | 0,10930 | poly(3-hydroxyalkanoic acid) synthase 2 |
| PA5100 | hutU | 2,5901 | 0,00854 | 0,05928 | urocanase |
| PA5106 | - | 2,7190 | 0,00484 | 0,03682 | conserved hypothetical protein |
| PA5180 | - | 6,0911 | 0,00768 | 0,03811 | conserved hypothetical protein |
| PA5181 | - | 4,8600 | 0,02241 | 0,09874 | probable oxidoreductase |
| PA5429 | aspA | 2,9749 | 0,00187 | 0,02989 | aspartate ammonia-lyase |
| PA5481 | - | 2,1058 | 0,01107 | 0,08797 | hypothetical protein |
| PA5506 | - | 2,7831 | 0,00204 | 0,02774 | hypothetical protein |
| PA5507 | - | 2,5467 | 0,00204 | 0,02859 | hypothetical protein |

**Table S3** Validation of selected genes from microarray analysis by qPCR.

| *gene* | expression | fold change | std. error |
| --- | --- | --- | --- |
| *nirS* | down | 0.03 | 0.014 - 0.056 |
| *nosZ* | down | 0,044 | 0.025 - 0.105 |
| *narK1* | down | 0.090 | 0.048 - 0.182 |
| *pvdH* | up | 6.583 | 2.415 - 17.199 |
| *pvdO* | up | 4.178 | 2.769 - 6.433 |
| *pvdL* | up | 26.438 | 6.78 - 118.09 |

*Experimental procedures*

*Quantification of mRNA by qPCR*

*P. aeruginosa* PAO1 and ΔsprP were grown in LB medium until cell growth reached an O.D. 580 nm of 2.5. RNA isolation was performed with an RNeasy minikit (Qiagen, Germany) according to the protocol of the manufacturer. Afterwards, genomic DNA was digested by DNase (Promega, United States). Quantitative reverse transcription-PCR (qRT-PCR) was performed using the standard instrument settings on an ABI 7900HT instrument using SYBR green fluorescence dye master mix. The expression levels were normalized to that of rpoD as a control housekeeping gene. Detection of mRNA of interest was achieved using specific primer (Tab. S4) and normalized to the level of the housekeeping gene rpoD. qPCR was analyzed using the relative expression software tool (REST) (Pfaffl *et al.*, 2002)

*Inactivation of SprP by site-specific mutagenesis*

The catalytic active serine was exchanged to alanine using QuickChange® site-directed mutagenesis according to the method of Edelheit *et. al.* 2009 using Phusion DNA Polymerase and up primer CGTCCCGACAAC**GCG**GGTTCGACGGTC and dn primer GACCGTCGAACC**CGC**GTTGTCGGG ACG.

**Table S4** Primer used for qPCR in this study

| gene name | Primer up | Primer dn |
| --- | --- | --- |
| *nirS* | GCTGTTCATCAAGACCCATC | GAGGTTCTTCAGGTCGAACA |
| *nosZ* | GCCTATACCACGCTGTTCAT | TACTGCACGTCGAGTTTCTG |
| *narK1* | ATCACCATGCTGTTCACCTT | GAGAGGAAGAACAGGCACAC |
| *pvdH* | GCAGTTGGTCAGCGTCTAT | TTGCAGACGAAGAAGATCAG |
| *pvdO* | ACTGGACGCCTACCTGAAG | GTCGTTGTAGTCGACGCATA |
| *pvdL* | ATCAAGGCGATCAAGGAAC | GTCGAACTGTCCGAGGTAGT |

*References*

**Edelheit, O., Hanukoglu, A., & Hanukoglu, I. (2009).** Simple and efficient site-directed mutagenesis using two single-primer reactions in parallel to generate mutants for protein structure-function studies. *BMC Biotechnol.* **9**, 61.

**Pfaffl M.W., Horgan G.W., and Dempfle L. (2002).** Relative expression software tool (REST (c)) for group-wise comparison and statistical analysis of relative expression results in real-time PCR. *Nucleic Acids Research*, **30** (9) e36
